# Urinary incontinence and quality of life: a systematic review and meta-analysis

**DOI:** 10.1007/s40520-020-01712-y

**Published:** 2020-09-22

**Authors:** Damiano Pizzol, Jacopo Demurtas, Stefano Celotto, Stefania Maggi, Lee Smith, Gabriele Angiolelli, Mike Trott, Lin Yang, Nicola Veronese

**Affiliations:** 1 Italian Agency for Development Cooperation, Khartoum, Sudan; 2 Clinical and Experimental Medicine PhD Program, University of Modena and Reggio Emilia, Modena, Italy; 3 Primary Care Department, USL Toscana Sud Est-Grosseto, Grosseto, Italy; 4 Primary Care Department, Azienda Sanitaria Universitaria Friuli Centrale, Udine, Italy; 5 National Research Council, Neuroscience Institute, Aging Branch, Padua, Italy; 6 Faculty of Sport Sciences, University of Murcia, Murcia, Spain; 7 Primary Care Department, Azienda Unità Locale Socio Sanitaria 3 “Serenissima”, Venice, Italy; 8 Department of Cancer Epidemiology and Prevention Research, Cancer Control Alberta, Alberta Health Services, Calgary, Canada; 9 Departments of Oncology and Community Health Sciences, University of Calgary, Calgary, Canada; 10 Geriatric Unit, Department of Internal Medicine and Geriatrics, University of Palermo, Palermo, Italy

**Keywords:** Quality of life, Urinary incontinence, Meta-analysis

## Abstract

**Background:**

Urinary incontinence (UI) and low quality of life (QoL) are two common conditions. Some recent literature proposed that these two entities can be associated. However, no attempt was made to collate this literature. Therefore, the aim of this study was to conduct a systematic review and meta-analysis of existing data to estimate the strength of the association between UI and QoL.

**Methods:**

An electronic search of major databases up to 18th April 2020 was carried out. Meta-analysis of cross-sectional and case–control studies comparing mean values in QoL between patients with UI and controls was performed, reporting random-effects standardized mean differences (SMDs) ± 95% confidence intervals (CIs) as the effect size. Heterogeneity was assessed with the *I*^2^.

**Results:**

Out of 8279 articles initially screened, 23 were finally included for a total of 24,983 participants, mainly women. The mean age was ≥ 50 years in 12/23 studies. UI was significantly associated with poor QoL as assessed by the short-form 36 (SF-36) total score (*n* = 6 studies; UI: 473 vs. 2971 controls; SMD = − 0.89; 95% CI − 1.3 to − 0.42; *I*^2^ = 93.5) and by the sub-scales of SF-36 and 5/8 of the domains included in the SF-36. Similar results were found using other QoL tools. The risk of bias of the studies included was generally high.

**Conclusions:**

UI is associated with a poor QoL, with a strong level of certainty. This work, however, mainly based on cross-sectional and case–control studies, highlights the necessity of future longitudinal studies for better understanding the importance of UI on QoL.

**Electronic supplementary material:**

The online version of this article (10.1007/s40520-020-01712-y) contains supplementary material, which is available to authorized users.

## Introduction

Urinary incontinence (UI) assumes an utmost importance in medicine, being a multifactorial syndrome defined as the sign of any involuntary leakage of urine [1–3]. UI is a widespread disorder affecting millions of people over the world with important and probably still underestimated negative consequences on personal and social wellbeing [4]. In particular, UI affects more females than males, even if female UI is yet often underestimated [4]. Although the exact prevalence is not known, at least one person out of four could be affected by UI during their life. [5, 6] UI due to chronic causes can be divided into five groups: urgency, stress, mixed, overflow and functional [7].

Regardless of its type, especially in older adults, UI is rarely  reported by the patient, because it is considered a natural consequence of ageing and, most of all, due to a sense of shame [8]. Often, affected individuals deny and hide UI, which results in physical and psychosocial restrictions to enjoyment in life. Actually, the key consequences include loss of self-confidence and social isolation in addition to other negative outcomes such as anxiety, depression, deterioration in sexual life and decrease in physical activity [9]. All these conditions are associated per se with poor quality of life (QoL), an umbrella term that, nowadays, includes various domains in human life that describes the expectations of an individual or society for a good life [10]. Despite increasing research in medicine indicating the importance of QoL and the high prevalence of UI in older adults, no attempt has yet been made to collate the literature investigating the association between UI and QoL in older adults in the attempt to derive a precise understanding on this topic.

Given this background, the aim of this study was to conduct a systematic review and meta-analysis of existing data to estimate the strength of the association between UI and QoL.

## Methods

This systematic review adhered to the PRISMA [11] and MOOSE [12] statements and followed a structured protocol submitted to PROSPERO (https://www.crd.york.ac.uk/prospero/display_record.php?RecordID=181768).

### Data sources and literature search strategy

Two investigators (NV and DP) independently conducted a literature search using MEDLINE/PubMed, Scopus, CINAHL, Embase PsycINFO and Cochrane Library databases from inception until 18th April 2020. Any inconsistencies were resolved by consensus with a third author (JD).

In PubMed, the following search strategy was used: “(urine incontinence OR bladder incontinence OR incontinence, urine OR urinary incontinence OR urinary leakage OR urine bladder incontinence OR urine incontinence OR urine leakage OR wetting, urine) AND (quality of life OR hrql OR health related quality of life OR life quality)”. Conference abstracts and reference lists of included articles were hand-searched to identify any potential additional relevant work.

### Study selection

Following the PICOS (participants, intervention, controls, outcomes, study design) criteria, we included studies assessing:

P: People with urinary incontinence
I: None
C: People without urinary incontinence
O: Quality of life
S: Observational (case–control, cross-sectional, cohort studies)

The diagnosis of UI could be made through self-reported information, through validated generic (e.g., Katz’s index [13]) or specific (e.g., Sandvik Severity Index [14]) questionnaires or instrumental tools (e.g., urodynamic tests); QoL was assessed through validated tools that are summarized in Supplementary Table 1. Studies were excluded if they included pediatric populations; if the data were not analyzable; or if they did not clearly report data regarding QoL tools in UI and/or controls. No language restriction was placed.

### Data extraction

For each eligible study, two independent investigators (NV, DP) extracted: name of the first author and year of publication, setting, sample size, mean age of the population, % of females, ethnicity, prevalence of some comorbidities related to urinary incontinence and QoL (such as % of dermatitis, % of disability and others), diagnostic tool used for QoL and for UI definitions, and the severity of the UI. These data were extracted, if possible, for those with UI and for controls, respectively. Data about matching and method (i.e., propensity score) were planned to be extracted, but no study included this information.

### Outcomes

The primary outcomes were considered the mean values and the correspondent standard deviations (SDs) of the validated tools of QoL, comparing the values of participants with UI and the controls. If the data were reported in other ways, e.g., median and interquartile ranges, they were transformed into means and SD.

### Assessment of study quality

Two independent authors (SC, JD) made the assessment of the studies’ quality using the Newcastle–Ottawa Scale (NOS) [15]. The NOS assigns a maximum of 9 points based on three quality parameters: selection, comparability, and outcome. As per the NOS grading in past reviews, we graded studies as having a high (< 5 stars), moderate (5–7 stars) or low risk of bias (≥ 8 stars) [16].

### Data synthesis and statistical analysis

All analyses were performed using Stata, version 15.0. For all analyses, a *p* value less than 0.05 was considered statistically significant.

The primary analysis compared the values of QoL tests between participants with UI vs. controls, according to the test used for assessing the QoL. We calculated the difference between the means of the UI and control groups through standardized mean differences (SMD) with their 95% confidence intervals (CIs), applying a random-effect model [17]. We then applied the indications for interpreting the magnitude of the SMD in the social sciences, as suggested by Cohen [18], i.e., small, SMD = 0.2–0.5; medium, SMD = 0.5–0.8; and large, SMD > 0.8. The data were also reported as forest plots, in a graphical way.

Heterogeneity across studies was assessed by the *I*^2^ metric. Given significant heterogeneity (*I*^2^ ≥ 50% and/or *p* < 0.05) [19] and having at least 10 studies for each outcome, we planned to run meta-regression analyses, taking as moderators the factors cited in the data extraction paragraph (see for more information Supplementary Table 2) in the sample as whole and as differences, in prevalence, between UI and controls. However, no outcome included 10 studies and so these analyses were not possible. Since the causes of UI are traditionally different between men and women, we assessed the percentage of women in each study as potential moderator of highly heterogeneous findings, having at least four studies for an outcome.

Publication bias was assessed by visual inspection of funnel plots and using the Egger bias test [20]. In case of publication bias, when ≥ 3 studies were available, we used the Duval and Tweedie non-parametric trim-and-fill method to account for potential publication bias [21]. Based on the assumption that the effect sizes of all the studies are normally distributed around the center of a funnel plot, in the event of asymmetries, this procedure adjusts for the potential effect of unpublished (trimmed) studies [20]. However, no outcome suffered on publication bias.

## Results

### Literature search

**Fig. 1:**
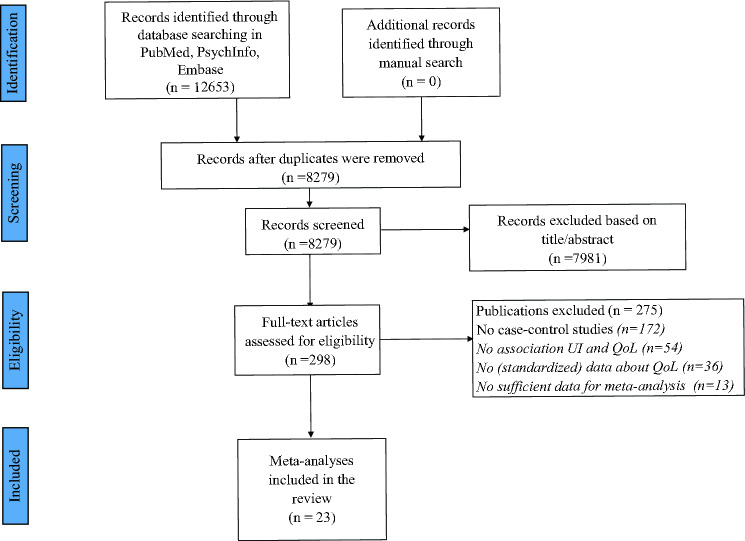
PRISMA flow-chart

As shown in Fig. 1, we initially found 8279 possible eligible articles. After removing 7981 works through the title/abstract screening, 298 were retrieved as full text. Of the 298 full text, 23 satisfied the inclusion/exclusion criteria and were, then, included in the systematic review and meta-analysis [22–44].

### Descriptive data

The 23 studies included a total of 24,983 participants (8723 with UI; 16,260 controls). The mean age was ≥ 50 years in 12/23 studies and were mainly women (only women: 14 studies; more than 50% women: 8 studies; only men: 1 study) (Table 1). The studies were mainly cross-sectional (14 cross-sectional and 9 case–control), made in America (8 studies) and mainly included outpatients (19 studies). UI was diagnosed mainly through self-reported information (18 studies). Only a few studies reported details regarding UI: namely 4 studies included only stress UI and 3 studies a mix of stress-urgency-mixed incontinence. Only one study reported the severity of UI (mild, moderate, severe) [29]. QoL was assessed mainly through short-form 36 (SF-36) [45] (10 studies), followed by other tools in order of frequency.

**Table 1:** Characteristics of the included studies

Author, year	Continent	Setting	Sample size	% women	Mean age of the population	Diagnostic tool urinary incontinence	Tool for quality of life	Severity/type of the UI	NOS
Aguilar-Navarro, 2012	America	Community	1124	58	79.5	Sandvik Severity Index	SF-36 (total score)	Mild–moderate-severe	6
Balkarli, 2016	Middle-east	Outpatient	157	89.2	46.0	Incontinence severity index	SF-36 (total score)	Not specified	4
Can, 2012	Middle-east	Outpatient	260	100	15–49	Self-reported	SF-36 (total score)	Not specified	2
Choi, 2014	Asia	Outpatient	519	55.9	62.5	Self-reported	IIQ-7	Stress-urge-mixed incontinence	4
Coyne, 2008	America	Outpatient	703	71.1	53.8	Self-reported	OABq-SF (HRQoL)	Not specified	3
de Mello Portella, 2011	America	Outpatient	147	100	52.3	Self-reported	SF-36 MCS + PCS	Not specified	4
De Nunzio, 2019	Europe	Outpatient	4596	0	64.5	ICIQ-UI	EORTC QLQ-C30	Not specified	4
de Oliveira, 2013	America	Outpatient	495	100	27.2	Self-reported	ICIQ-SF	Not specified	6
Duggan, 2011	Oceania	Outpatient	139	100	58.1	Urodynamic	King’s Health Questionnaire	Stress incontinence	7
Gascon, 2018	America	Outpatient	31	67.5	51.7	Self-reported	King’s Health Questionnaire	Not specified	3
Goris, 2010	Middle-east	Outpatient	48	100	35.9	Self-reported	SF-36 (total score)	Not specified	3
Hawkins, 2010	America	Community	5530	58.2	NA	Self-reported	SF-36 MCS + PCS	Not specified	7
Horng, 2012	Asia	Community	4661	100	49.5	Self-reported	SF-36 MCS + PCS	Not specified	8
Lim, 2016	Asia	Outpatient	265	100	50.0	Self-reported	ICIQ-LUTSquol	Not specified	6
Lin, 2018	Asia	Outpatient	560	100	32.2	Self-reported	IIQ-7	Not specified	3
Mallah, 2013	Middle-east	Outpatient	140	100	43.2	Self-reported	GHQ-12	Stress-urge-mixed incontinence	3
Martínez Agulló, 2010	Europe	Outpatient and nursing home	893	88.4	80.2	Self-reported	SF-36 MCS + PCS	UI + OAB	3
Oh, 2006	Asia	Outpatient	228	100	NA (for control group)	Self-reported	SF-36 MCS + PCS	Stress incontinence	3
Rannestad, 2011	Europe	Outpatient	653	100	52.5	International Continence Society	Ferrans & Powers’ QLI	Not specified	6
Schimpf, 2009	America	Outpatient	465	100	NA	Self-reported	PFIQ	Stress incontinence	5
Steibliene, 2020	Europe	Outpatient	177	100	50.2	Self-reported	King’s Health Questionnaire	Stress incontinence	6
Tang, 2013	America	Outpatient	1607	76	60.7	Self-reported	EQ-5D utility	Not specified	6
Tozun, 2009	Middle-east	Outpatient	1585	100	≥ 60 years: 22.3%	Self-reported	SF-36 MCS + PCS	Stress-urge-mixed incontinence	5
Total	America: 8 studies; Middle-East 5 studies; Asia: 5 studies; Europe: 4 studies; Oceania: 1 study	Outpatients: 19 studies; community: 3 studies; community and nursing home: 1 study	24,983	Only women: 14 studies; more than 50% women; 8 studies; only men: 1 study	Mean age ≥ 50 years: 12 studies; mean age < 50 years: 8 studies: not available: 3 studies	Self-reported: 18 studies; urodynamic: 1 study; other tools: 4 studies	SF-36: 10 studies; King’s Health Questionnaire: 3 studies; other tools: 10 studies	Not specified: 14 studies; only stress incontinence: 4 studies; stress-urge-mixed incontinence: 3 studies; different degrees of UI: 1 study; others: 1 study	Median = 4 (range 2–8)

*EORTC QLQ-C30* European Organization for Research and Treatment of Cancer Quality of Life Questionnaire Version 3.0, *EQ-5D utility* EuroQuol 5 domains, *GHQ-12* General Health Questionnaire 12-items, *ICIQ-LUTSquol* International Consultation on Incontinence Questionnaire Lower Urinary Tract Symptoms Quality of Life, *IIQ-7* Incontinence Impact Questionnaire, short-form 7-items, *NOS* Newcastle–Ottawa quality assessment Scale, *PFIQ* Pelvic Floor Impact Questionnaire, *QoL* quality of life, *SF-36* 36-Item Short-Form Survey, *UI* urinary incontinence

### Urinary incontinence and quality of life

**Fig. 2:**
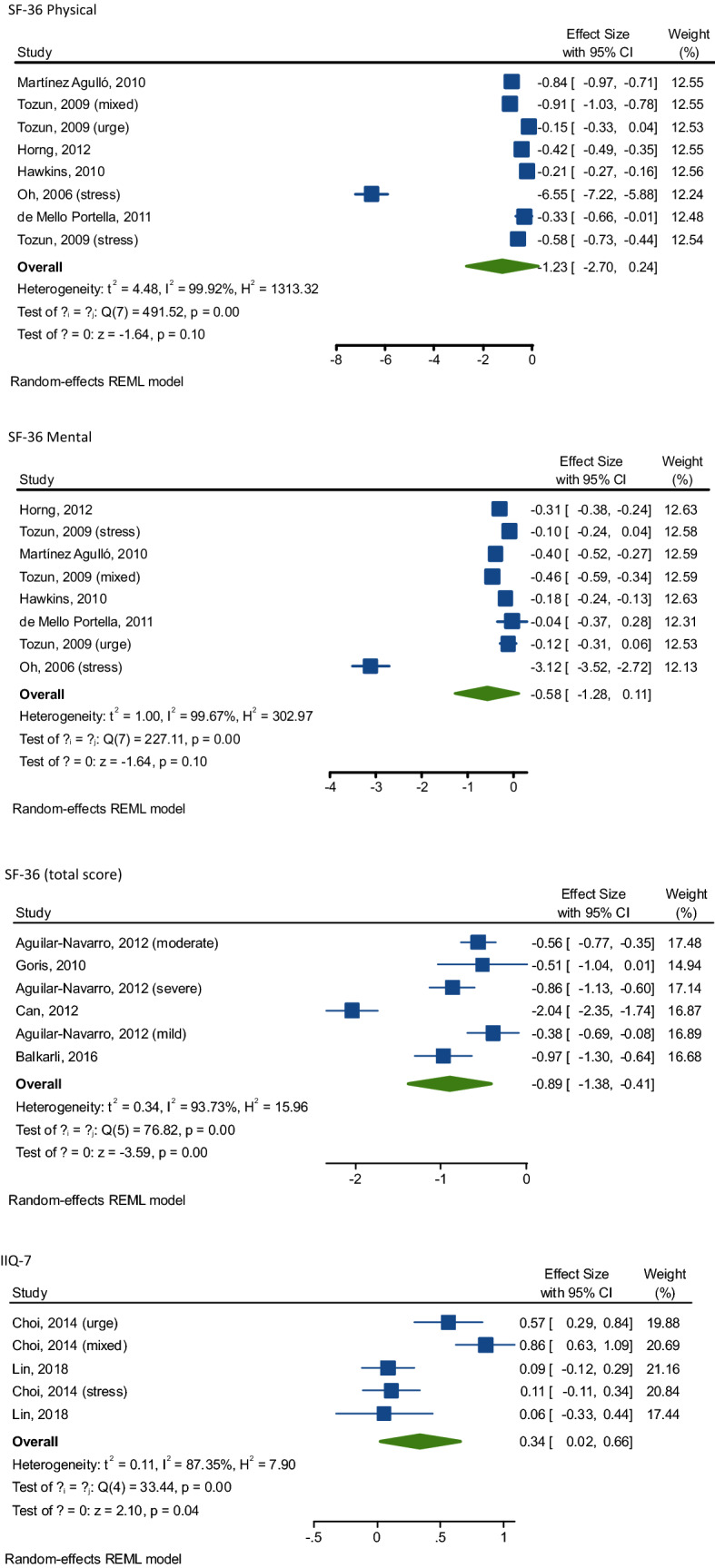

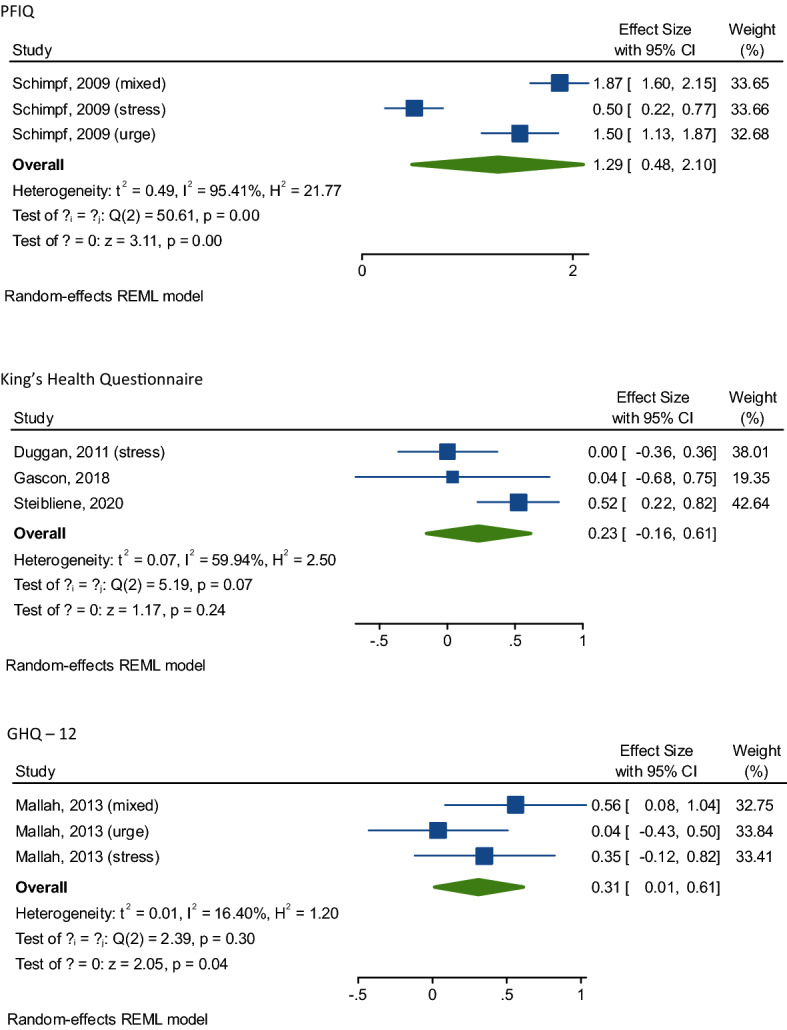
Association of urinary incontinence with quality of life, effect sizes represented in standard mean difference (SMD) and 95% confidence intervals

As shown in Tables 2 and 3 and graphically in Fig. 2, UI was significantly associated with poor QoL. For example, for the SF-36 (total score), we observed in six studies (UI: 473 vs. 2971 controls) a SMD = − 0.89 (95% CI − 1.3 to − 0.42; *I*^2^ = 93.5), indicating a large association between UI and low QoL comparing to controls. The same results presented for SF-36 sub-scales, i.e., for SF-36 mental and for SF-36 physical scores, where the association was medium and large, respectively, in 8 studies including 4604 participants with UI and 10,121 controls.

**Table 2:** Main findings regarding quality of life (QoL) and urinary incontinence (UI) using short-form 36

Tools for QoL	Number of comparisons	UI	Controls	SMD	95% CI		*p* value	*I*^2^	Egger’s test (*p* value)
SF-36 (total score)	6	473	2971	− 0.89	− 1.3	− 0.42	< 0.0001	93.5	− 10.0 ± 4.27 (0.06)
SF-36 Mental	8	4604	10,121	− 0.52	− 0.75	− 0.29	< 0.0001	96.9	− 2.29 ± 7.08 (0.76)
SF-36 Physical	8	4604	10,121	− 1.04	− 1.39	− 0.69	< 0.0001	98.6	− 5.17 ± 3.61 (0.20)
General health perception	8	4604	10,121	− 0.76	− 1.00	− 0.52	< 0.0001	94.8	− 15.1 ± 7.27 (0.26)
Physical function	7	4392	9835	− 1.04	− 1.46	− 0.63	< 0.0001	97.9	− 6.28 ± 12.27 (0.96)
Social function	7	4392	9835	− 0.44	− 0.71	− 0.18	0.001	95	− 5.42 ± 8.45 (0.48)
Phys role function	7	4392	9835	− 0.91	− 1.88	0.07	0.07	99.6	− 12.54 ± 8.27 (0.46)
Emotional role function	7	4392	9835	0.07	− 0.02	0.34	0.61	95.1	8.21 ± 8.24 (0.96)
Mental health	7	4392	9835	− 0.58	− 0.88	− 0.27	< 0.0001	96.3	− 4.56 ± 4.68 (0.98)
Vitality	7	4392	9835	− 0.50	− 0.76	− 0.24	< 0.0001	94.9	− 8.45 ± 8.27 (0.95
Pain	7	4392	9835	− 0.21	− 0.52	0.10	0.19	96.4	− 2.54 ± 2.27 (0.96)

*CI* confidence interval, *EORTC QLQ-C30* European Organization for Research and Treatment of Cancer Quality of Life Questionnaire Version 3.0, *EQ-5D utility* EuroQuol 5 domains, *GHQ-12* General Health Questionnaire 12-items, *ICIQ-LUTSquol* International Consultation on Incontinence Questionnaire Lower Urinary Tract Symptoms Quality of Life, *IIQ-7* Incontinence Impact Questionnaire, short-form 7-items, *PFIQ* Pelvic Floor Impact Questionnaire, *QoL* quality of life, *SF-36* 36-Item Short-Form Survey, *SMD* standardised mean difference, *UI* urinary incontinence

**Table 3:** Main findings regarding quality of life (QoL) and urinary incontinence (UI) using other tools for assessing QoL

Tools for QoL	Number of comparisons	UI	Controls	SMD	95% CI		*p* value	*I*^2^	Egger’s test (*p* value)
IIQ-7	5	474	1279	0.34	0.01	0.67	0.04	88	0.07 ± 7.88 (0.99)
EORTC QLQ-C30	1	771	3825	0.20	0.12	0.28	< 0.0001	–	–
King’s Health Questionnaire	3	196	151	0.23	− 0.17	0.62	0.26	61.5	− 2.32 ± 3.60 (0.64)
ICIQ-LUTSquol	1	120	145	2.52	2.20	2.85	< 0.0001	–	–
GHQ-12	3	105	105	0.31	0.01	0.61	0.04	16.3	107 ± 26 (0.15)
Ferrans & Powers’	1	225	428	− 6.29	− 6.67	− 5.92	< 0.0001	–	–
PFIQ	3	354	333	1.29	0.41	2.16	0.004	96.0	7.32 ± 31.38 (0.85)
EQ-5D utility	1	907	700	− 0.38	− 0.48	− 0.28	< 0.0001	–	–

*CI* confidence interval, *EORTC QLQ-C30* European Organization for Research and Treatment of Cancer Quality of Life Questionnaire Version 3.0, *EQ-5D utility* EuroQuol 5 domains, *GHQ-12* General Health Questionnaire 12-items, *ICIQ-LUTSquol* International Consultation on Incontinence Questionnaire Lower Urinary Tract Symptoms Quality of Life, *IIQ-7* Incontinence Impact Questionnaire, short-form 7-items, *PFIQ* Pelvic Floor Impact Questionnaire, *QoL* quality of life, *SF-36* 36-Item Short-Form Survey, *SMD* standardised mean difference, *UI* urinary incontinence

When assessing the singular domain of the SF-36, UI was associated with significant worse scores in general health perception, physical and social function, mental health and vitality, with a medium–large strength of these associations (Table 2).

Similar findings were presented when using the Incontinence Impact Questionnaire (IIQ-7) [46] in five studies, including 474 participants with UI and 1279 controls. Using this tool, the SMD was 0.34 (95% CI 0.01–0.67; *I*^2^ = 88%) (Table 3, Fig. 2). The percentage of women in the studies was not associated with worse QoL, in meta-regression analysis.

Finally, statistically significant results were found when using other tools for assessing QoL, even if these outcomes included ≤ 3 studies (Table 3, Fig. 2).

### Publication bias

As fully reported in Table 1, no included outcome suffered on publication bias.

### Risk of bias

The risk of bias, evaluated through the NOS, was fully reported in Table 1 (as total score) and Supplementary Table 3 (for case–control studies) and 4 (for cross-sectional studies), respectively. Six case–control studies over 9 suffered on low quality (high risk of bias) as indicated by a NOS < 5/9. The selection and the representativeness of cases and controls were predominant problems in these studies (Supplementary Table 3).

Furthermore, half of the cross-sectional studies suffered on potential high risk of bias. Again, issues regarding the sample size definition, poor descriptions of non-respondent and lack of matching were the main shortcomings for these studies (Supplementary Table 4).

## Discussion

In this systematic review and meta-analysis, including 23 studies and 24,983 participants (8723 with UI; 16,260 controls), we found that the presence of UI was significantly associated with poor QoL. These results, even if characterized by a high heterogeneity and a potential high risk of bias, are of importance, since they add new insight regarding this important topic.

Previously, approximately 10-years ago at the time of writing, two systematic reviews without any formal meta-analysis reached similar conclusions. One review [47] reported that women with UI had lower QoL than their counterparts, but the findings were limited by the small sample size included; the other systematic review [48] found that overactive bladder can be associated with lower QoL levels, but did not include any other UI type. The present meta-analysis overcomes these inherent limitations. First, this review included both men and women. Even if UI is a typical condition of women [49], increasing research is showing the importance of UI in men [50]. Second, all types of UI and not only overactive bladder were included in the present review. Finally, this work incorporated a meta-analytic approach, quantifying the possible association between UI and QoL showing that UI is associated with a poor QoL with a strong/medium strength.

UI may be associated with poor QoL via several mechanisms. First, people with UI usually exhibit more comorbidities than those without. Although several risk factors are reported, the most specifically related are sex, age, dementia, and mobility ability [51]. In addition, fluid intake, self-mobility, diuretic treatment may also influence diuresis and thus UI [51]. It is widely known that all these factors are associated with poor QoL in older people. We have tried to explore the role of comorbidities for explaining our findings, but, unfortunately, no sufficient data are presented in the studies included, as shown in Supplementary Table 2. Second, it is possible that people having UI can use diapers and the use of these tools can lead, in particular conditions, to the Incontinence-Associated Dermatitis (IAD) [52]. IAD, as other dermatological conditions, is associated with a poor QoL [53]: unfortunately, no one of the 23 studies included, reported data regarding this important condition that should be explored in future studies. Finally, we believe that poor QoL in UI can be justified by the presence of shame in these people leading to a change in their lifestyle and habits [54] (i.e., reduction or suppression of physical activity) and to a development of mental disorders (i.e., depression [54] and anxiety [55]). This could be particularly true in younger people [8].

Finally, as reported in a systematic review published over a decade ago [56], only a few interventions are able to improve QoL in people affected by UI. Across 96 randomized controlled trials included, one study including 451 women reported that duloxetine significantly improved QoL compared to placebo [57], whilst pelvic-floor muscle exercise position did not affect QoL in patients affected by UI [58]. These findings suggest that more research is needed for better understanding pharmacological and non-pharmacological interventions able to improve QoL in UI.

The findings of our study should be interpreted within its limitations. First, only case–control and cross-sectional studies were included, and these studies have inherent limitations, potentially introducing a reverse bias (i.e., people with poor QoL for other reasons may experience UI). Second, the included studies mainly encompassed women as participants, but UI is an important condition also in men: the results of this study may thereby not be directly applicable to a male population. Third, the results were highly heterogeneous and thus it is not possible to explain this issue through a meta-regression, since the data reported for the moderators and planned in our protocol are too inconclusive. Finally, several studies are at high risk of bias. Of importance, no study preformed matching between participants with UI and controls, potentially introducing a bias.

In conclusion, the present systematic review and meta-analysis showed that UI is associated with a poorer QoL when compared to controls, with a strong level of certainty. This work, mainly based on cross-sectional and case–control studies at high risk of bias, highlights the necessity of future longitudinal studies for better understanding the importance of UI in determining QoL.

## Electronic supplementary material

Below is the link to the electronic supplementary material.

Supplementary material 1 (DOCX 30 kb)

## References

[1] Aharony L, De Cock J, Nuotio M (2017). Consensus document on the management of urinary incontinence in older people. Eur Geriatr Med.

[2] Abrams P, Cardozo L, Fall M (2002). The standardisation of terminology of lower urinary tract function: report from the Standardisation Sub-committee of the International Continence Society. Am J Obstet Gynecol.

[3] Veronese N, Soysal P, Stubbs B (2018). Association between urinary incontinence and frailty: a systematic review and meta-analysis. Eur Ger Med.

[4] Dooley Y, Kenton K, Cao G (2008). Urinary incontinence prevalence: results from the National Health and Nutrition Examination Survey. J Urol.

[5] Stewart WF, Hirsh AG, Kirchner HL (2014). Urinary incontinence incidence: quantitative meta-analysis of factors that explain variation. J Urol.

[6] Ebbesen MH, Hunskaar S, Rortveit G (2013). Prevalence, incidence and remission of urinary incontinence in women: longitudinal data from the Norwegian HUNT study (EPINCONT). BMC Urol.

[7] Gibbs CF, Johnson TM, Ouslander JG (2007). Office management of geriatric urinary incontinence. Am J Med.

[8] Elenskaia K, Haidvogel K, Heidinger C (2011). The greatest taboo: urinary incontinence as a source of shame and embarrassment. Wien Klin Wochen.

[9] Farage MA, Miller KW, Berardesca E (2008). Psychosocial and societal burden of incontinence in the aged population: a review. Arch Gynecol Obstet.

[10] Guyatt GH, Feeny DH, Patrick DL (1993). Measuring health-related quality of life. Ann Int Med.

[11] Liberati A, Altman DG, Tetzlaff J (2009). The PRISMA statement for reporting systematic reviews and meta-analyses of studies that evaluate health care interventions: explanation and elaboration. PLoS Med.

[12] Stroup DF, Ja Berlin, Morton SC (2000). Meta-analysis of observational studies in epidemiology: a proposal for reporting. Meta-analysis Of Observational Studies in Epidemiology (MOOSE) group. JAMA.

[13] Katz S, Downs TD, Cash HR (1970). Progress in development of the index of ADL. Gerontologist.

[14] Sandvik H, Hunskaar S, Seim A (1993). Validation of a severity index in female urinary incontinence and its implementation in an epidemiological survey. J Epidemiol Community Health.

[15] Wells GA, Tugwell P, O’Connell D et al (2015) The Newcastle–Ottawa Scale (NOS) for assessing the quality of nonrandomized studies in meta-analyses. http://www.ohri.ca/programs/clinical_epidemiology/oxford.asp. Accessed 1 June 2020

[16] Luchini C, Stubbs B, Solmi M (2017). Assessing the quality of studies in meta-analysis: advantages and limitations of the Newcastle Ottawa Scale. World J Meta-Anal.

[17] Higgins JPT, Green S (2008). Cochrane handbook for systematic reviews.

[18] Cohen J (1992). Statistical power analysis. Curr Dir Psychol Sci.

[19] Higgins JP, Altman DG, Gotzsche PC (2011). The Cochrane Collaboration’s tool for assessing risk of bias in randomised trials. BMJ.

[20] Egger M, Davey Smith G, Schneider M (1997). Bias in meta-analysis detected by a simple, graphical test. BMJ.

[21] Duval S, Tweedie R (2000). A nonparametric “trim and fill” method of accounting for publication bias in meta-analysis. J Am Stat Assoc.

[22] Oh SJ, Ku JH (2007). Impact of stress urinary incontinence and overactive bladder on micturition patterns and health-related quality of life. Int Urogynecol J Pelvic Floor Dysfunct.

[23] Coyne KS, Sexton CC, Irwin DE (2008). The impact of overactive bladder, incontinence and other lower urinary tract symptoms on quality of life, work productivity, sexuality and emotional well-being in men and women: results from the EPIC study. BJU Int.

[24] Schimpf MO, Patel M, O’Sullivan DM (2009). Difference in quality of life in women with urge urinary incontinence compared to women with stress urinary incontinence. Int Urogynecol J Pelvic Floor Dysfunct.

[25] Tozun M, Ayranci U, Unsal A (2009). Prevalence of urinary incontinence among women and its impact on quality of life in a semirural area of Western Turkey. Gynecol Obstet Investig.

[26] Martínez Agulló E, Ruíz Cerdá JL, Gómez Pérez L (2010). Impact of urinary incontinence and overactive bladder syndrome on health-related quality of life of working middle-aged patients and institutionalized elderly patients. Act Urol Españ (Engl Ed).

[27] Duggan P (2011). Urodynamic diagnoses and quality of life in women presenting for evaluation of urinary incontinence. Aust N Z J Obstet Gynaecol.

[28] Rannestad T, Skjeldestad FE (2011). Ferrans and Powers’ Quality of life index applied in urinary incontinence research—a pilot study. Scand J Caring Sci.

[29] Aguilar-Navarro S, Navarrete-Reyes AP, Grados-Chavarria BH (2012). The severity of urinary incontinence decreases health-related quality of life among community-dwelling elderly. J Gerontol A Biol Sci Med Sci.

[30] de Mello Portella P, Feldner PC, da Conceicao JC (2012). Prevalence of and quality of life related to anal incontinence in women with urinary incontinence and pelvic organ prolapse. Eur J Obstet Gynecol Reprod Biol.

[31] Horng SS, Huang N, Wu SI (2013). The epidemiology of urinary incontinence and it’s influence on quality of life in Taiwanese middle-aged women. Neurourol Urodyn.

[32] Oliveira CD, Seleme M, Cansi PF (2013). Urinary incontinence in pregnant women and its relation with socio-demographic variables and quality of life. Rev Ass Méd Bras (Engl Ed).

[33] Choi EP, Lam CL, Chin WY (2014). The health-related quality of life of Chinese patients with lower urinary tract symptoms in primary care. Qual Life Res.

[34] Tang DH, Colayco DC, Khalaf KM (2014). Impact of urinary incontinence on healthcare resource utilization, health-related quality of life and productivity in patients with overactive bladder. BJU Int.

[35] Lim R, Liong ML, Leong WS (2016). Effect of stress urinary incontinence on the sexual function of couples and the quality of life of patients. J Urol.

[36] Gascon MRP, Mellao MA, Mello SH (2018). The impact of urinary incontinence on the quality of life and on the sexuality of patients with HAM/TSP. Braz J Infect Dis.

[37] Lin YH, Chang SD, Hsieh WC (2018). Persistent stress urinary incontinence during pregnancy and one year after delivery; its prevalence, risk factors and impact on quality of life in Taiwanese women: an observational cohort study. Taiwan J Obstet Gynecol.

[38] De Nunzio C, Pastore AL, Lombardo R (2019). The EORTC quality of life questionnaire predicts early and long-term incontinence in patients treated with robotic assisted radical prostatectomy: analysis of a large single center cohort. Urol Oncol.

[39] Steibliene V, Aniuliene R, Aniulis P (2020). Affective symptoms and health-related quality of life among women with stress urinary incontinence: cross-sectional study. Neuropsychiatr Dis Treat.

[40] Xiao J, Caan BJ, Weltzien E (2018). Associations of pre-existing co-morbidities with skeletal muscle mass and radiodensity in patients with non-metastatic colorectal cancer. J Cachexia Sarcopenia Muscle.

[41] Hawkins K, Pernarelli J, Ozminkowski RJ (2011). The prevalence of urinary incontinence and its burden on the quality of life among older adults with medicare supplement insurance. Qual Life Res.

[42] Balkarli A, Semiz M, Uslu AU (2016). An assessment of sleep disturbances and quality of life in primary Sjögren’s syndrome and its relationship with urinary incontinence. Acta Med Mediter.

[43] Goris S, Sungur G, Tasci S et al (2010) The effect of urinary incontinence and sexual dysfunction on the quality of life among women with multiple sclerosis. Pakistan J Med Sci 26:277–281.

[44] Mallah F, Montazeri A, Ghanbari Z (2014). Effect of urinary incontinence on quality of life among Iranian women. J Fam Reprod Health.

[45] Jenkinson C, Coulter A, Wright L (1993). Short form 36 (SF36) health survey questionnaire: normative data for adults of working age. BMJ.

[46] Moore KN, Jensen L (2000). Testing of the Incontinence Impact Questionnaire (IIQ-7) with men after radical prostatectomy. J WOCN.

[47] Kwon BE, Kim GY, Son YJ (2010). Quality of life of women with urinary incontinence: a systematic literature review. Int Neurourol.

[48] Bartoli S, Aguzzi G, Tarricone R (2010). Impact on quality of life of urinary incontinence and overactive bladder: a systematic literature review. Urology.

[49] Norton P, Brubaker L (2006). Urinary incontinence in women. Lancet.

[50] Markland AD, Goode PS, Redden DT (2010). Prevalence of urinary incontinence in men: results from the national health and nutrition examination survey. J Urol.

[51] Offermans MP, Du Moulin MF, Hamers JP (2009). Prevalence of urinary incontinence and associated risk factors in nursing home residents: a systematic review. Neurourol Urodyn.

[52] Beele H, Smet S, Van Damme N (2018). Incontinence-associated dermatitis: pathogenesis, contributing factors, prevention and management options. Drugs Aging.

[53] Beeckman D, Campbell J, Campbell K (2015). Incontinence-associated dermatitis: moving prevention forward. Wounds Int.

[54] Nygaard I, DeLancey J, Arnsdorf L (1990). Exercise and incontinence. Obs Gyn.

[55] Bogner HR, Gallo JJ, Swartz KL (2002). Anxiety disorders and disability secondary to urinary incontinence among adults over age 50. Int J Psychiatry Med.

[56] Shamliyan TA, Kane RL, Wyman J (2008). Systematic review: randomized, controlled trials of nonsurgical treatments for urinary incontinence in women. Ann Intern Med.

[57] Kinchen KS, Obenchain R, Swindle R (2005). Impact of duloxetine on quality of life for women with symptoms of urinary incontinence. Int Urogynecol J.

[58] Borello-France DF, Zyczynski HM, Downey PA (2006). Effect of pelvic-floor muscle exercise position on continence and quality-of-life outcomes in women with stress urinary incontinence. Phys Ther.

