# Supplementary material for: Urinary incontinence and quality of life: a systematic review and meta-analysis
Source: Aging Clin Exp Res. 2020 Sep 22;33(1):25–35. doi: 10.1007/s40520-020-01712-y (PMC7897623; doi:10.1007/s40520-020-01712-y)
Supplement: Supplementary file 1 — Supplementary material 1 (DOCX 30 kb) [file 40520_2020_1712_MOESM1_ESM.docx]

**Supplementary Table 1. Summary for the tools assessing quality of life in the studies included**

| **Tools for QoL** | **Psychometric properties** | **Scores and interpretation** | **Higher scores indicate** |
| --- | --- | --- | --- |
| **SF-36 (total score)** | Eight domains: physical functioning (PF), role physical (RP), bodily pain (BP), general health (GH), vitality (VT), social functioning (SF), role emotional (RE), and mental health (MH). | 0-100  The authors reported that global score does not make sense | Higher quality of life |
| **SF-36 Mental** | The score is derived from: vitality (VT), social functioning (SF), role emotional (RE), and mental health (MH). | 0-100 | Higher quality of life |
| **SF-36 Physical** | The score is derived from: physical functioning (PF), role physical (RP), bodily pain (BP), general health (GH), | 0-100 | Higher quality of life |
| **IIQ-7** | Seven domains: household chores, physical recreation, entertaining activities, ability to travel, social activities, emotional health, feeling frustrated | 0 - 21 | Lower quality of life |
| **EORTC QLQ-C30** | Global health status, functional scales and symptom scales | 0 - 100 | Higher quality of life |
| **King’s Health Questionnaire** | Ten domains: general health, incontinence impact, symptoms, severity changes in health, physical function, role limitation, social limitation, mental health, energy/vitality. | 0 - 100 | Lower quality of life |
| **ICIQ-LUTSquol** | 20 items: household tasks, job, physical activities, travel, social life, friends, relationships, sex life, family, depression, anxiety, bad feelings, sleep, tired, pad use, fluid restriction, clothing issues, smell, embarrassment overall impact. | 19 - 76 | Higher quality of life |
| **GHQ - 12** | 12 items investigating social dysfunction, anxiety, and loss of confidence | 0 - 36 | Lower quality of life |
| **Ferrans & Powers’** | Five scores: QoL overall, health and functioning, socioeconomic, psychological/spiritual, and family | 0 - 30 | Higher quality of life |
| **PFIQ** | Seven domains: household chores, physical activities, entertainment activities, ability to travel, social activities, emotional health, feeling frustrated | 0 - 100 | Lower quality of life |
| **EQ-5D utility** | Five dimensions: mobility, self-care, usual activities, pain/discomfort and anxiety/depression | 0 -100 | Higher quality of life |

**Abbreviations:** EORTC QLQ-C30 = European Organization for Research and Treatment of Cancer Quality of Life Questionnaire Version 3.0; EQ-5D utility = EuroQuol 5 domains; GHQ - 12 = General Health Questionnaire 12-items; ICIQ-LUTSquol = International Consultation on Incontinence Questionnaire Lower Urinary Tract Symptoms Quality of Life; IIQ-7 = Incontinence Impact Questionnaire, short form 7-items; PFIQ = Pelvic Floor Impact Questionnaire; QoL = quality of life; SF-36 = 36-Item Short Form Survey

**Supplementary Table 2. Prevalence of major comorbidities in the overall sample in the studies included.**

| **Author. year** | **% of diabetes** | **% hypertension** | **% with disability** | **% with cancer** | **% heart disease** | **% with CVD** | **% osteoarthritis** | **% obese** | **Mean BMI** | **Current Smoking %** | **Depression %** | **% dermatitis** | **% fecal incontinence** |
| --- | --- | --- | --- | --- | --- | --- | --- | --- | --- | --- | --- | --- | --- |
| Aguilar-Navarro. 2012 | NA | 73.2 | 10.2 | NA | NA | NA | 51.2 | NA | NA | 48.5 | 30.2 | NA | NA |
| Balkarli. 2016 | NA | NA | NA | NA | NA | NA | NA | NA | NA | NA | NA | NA | NA |
| Can. 2012 | NA | NA | NA | NA | NA | NA | NA | NA | NA | NA | NA | NA | NA |
| Choi. 2014 | 20.4 | 59.5 | NA | 3.5 | 11.2 | NA | NA | NA | NA | 2.9 | 17.7 | NA | NA |
| Coyne. 2008 | 7.7 | 27 | NA | NA | NA | NA | NA | NA | NA | NA | 10.7 | NA | NA |
| de Mello Portella. 2011 | NA | NA | NA | NA | NA | NA | NA | NA | NA | NA | NA | NA | NA |
| De Nunzio. 2019 | NA | NA | NA | NA | NA | NA | NA | NA | 27 | NA | NA | NA | NA |
| de Oliveira. 2013 | NA | NA | NA | NA | NA | NA | NA | NA | NA | NA | NA | NA | NA |
| Duggan. 2011 | NA | NA | NA | NA | NA | NA | NA | NA | NA | NA | NA | NA | NA |
| Goris. 2010 | NA | NA | NA | NA | NA | NA | NA | NA | NA | NA | NA | NA | NA |
| Hawkins. 2010 | 18.5 | 61.3 | NA | 19.4 | 7.1 | 7.1 | 38.1 | 20.2 | NA | NA | NA | NA | NA |
| Horng. 2012 | 5.6 | 14.6 | NA | NA | 5.4 | 0.7 | NA | 16.6 | NA | 6.7 | NA | NA | NA |
| Gascon. 2018 | NA | NA | NA | NA | NA | NA | NA | NA | NA | NA | 41.9 | NA | NA |
| Lim. 2016 | NA | NA | NA | NA | NA | NA | NA | NA | NA | NA | NA | NA | NA |
| Lin. 2018 | NA | NA | NA | NA | NA | NA | NA | NA | 26.4 | NA | NA | NA | NA |
| Mallah. 2013 | NA | NA | NA | NA | NA | NA | NA | NA | NA | NA | NA | NA | NA |
| Oh. 2006 | NA | NA | NA | NA | NA | NA | NA | NA | NA | NA | NA | NA | NA |
| Rannestad. 2011 | NA | NA | NA | NA | NA | NA | NA | NA | NA | NA | NA | NA | NA |
| Schimpf. 2009 | NA | NA | NA | NA | NA | NA | NA | NA | NA | NA | NA | NA | NA |
| Steibliene. 2020 | NA | NA | NA | NA | NA | NA | NA | NA | NA | NA | NA | NA | NA |
| Tang. 2013 | NA | NA | NA | NA | NA | NA | NA | NA | NA | NA | NA | NA | NA |
| Tozun. 2009 | 9.2 | NA | NA | NA | NA | 0.9 | NA | NA | 26.2 | 21.3 | 14.1 | NA | NA |
| Martínez Agulló. 2010 | NA | NA | NA | NA | NA | NA | NA | NA | NA | NA | NA | NA | NA |
| **Total** | **12.3** | **47.1** | **10.2** | **19.4** | **7.9** | **2.9** | **44.6** | **18.4** | **26.5** | **10.3** | **21.1** | **no data** | **no data** |

**Abbreviations:** BMI = body mass index, CVD = cerebro vascular disease

**Supplementary Table 3. Quality assessment of case-control studies assessed with the Newcastle Ottawa Scale.**

| **Study, year** | **Is the case definition adequate?** | **Representativeness of the**  **cases** | **Selection of Controls** | **Definition of Controls** | **Comparability of cases and controls on the basis of the design or analysis** | **Ascertainment of exposure** | **Same method of ascertainment for cases and controls** | **Non-Response rate** | **Tot** |
| --- | --- | --- | --- | --- | --- | --- | --- | --- | --- |
| **Coyne 2008** | * | - | - | * | *- | - | - | - | 3 |
| **De Nunzio 2019** | - | * | - | * | *- | * | - | - | 4 |
| **De Mello Portella 2012** | - | - | * | * | ** | - | - | - | 4 |
| **Duggan 2011** | * | * | - | - | ** | * | * | * | 7 |
| **Lim 2016** | * | - | - | * | ** | - | * | * | 6 |
| **Lin 2018** | * | - | - | - | ** | - | - | - | 3 |
| **Oh 2006** | - | - | - | * | *- | - | * | - | 3 |
| **Rannestad 2010** | * | - | - | * | ** | - | * | * | 6 |
| **Schimpf 2009** | * | - | - | * | ** | - | * | - | 5 |

Note: A study can be awarded a maximum of one star for each numbered item within the Selection and Exposure categories. A maximum of two stars can be given for Comparability.

**Supplementary Table 4. Quality assessment of cross-sectional studies assessed with the Newcastle Ottawa Scale.**

| **Study, year** | **Representativeness of the sample** | **Sample size** | **Non-respondents** | **Ascertainment of the exposure** | **Comparability of cases and controls on the basis of the design or analysis** | **Assessment of the outcome** | **Statistical test** | **Tot** |
| --- | --- | --- | --- | --- | --- | --- | --- | --- |
| **Aguilar Navarro 2012** | * | - | - | ** | * | * | * | 6 |
| **Balkarli 2016** | - | - | - | ** | - | * | * | 4 |
| **Can 2012** | - | - | - | - | * | - | * | 2 |
| **Choy 2014** | * | - | - | * | - | * | * | 4 |
| **De Oliveira 2013** | * | * | - | * | * | * | * | 6 |
| **Gascon 2018** | - | - | * | * | - | - | * | 3 |
| **Goris 2010** | - | - | - | * | - | * | * | 3 |
| **Hawkins 2010** | * | * | * | * | * | * | * | 7 |
| **Horng 2012** | * | * | * | ** | * | * | * | 8 |
| **Mallah 2013** | - | - | - | * | - | * | * | 3 |
| **Martinez-Agullò 2010** | - | - | - | * | - | * | * | 3 |
| **Steibliene 2020** | * | - | - | ** | * | * | * | 6 |
| **Tang 2014** | * | * | - | ** | - | * | * | 6 |
| **Tozun 2009** | * | - | - | ** | - | * | * | 5 |

Note: A study can be awarded a maximum of one star for each numbered item within the Selection and Exposure categories. A maximum of two stars can be given for Comparability.
